# Seroprevalence of Hepatitis E among Iranian Renal Transplant Recipients

**DOI:** 10.5812/kowsar.1735143X.690

**Published:** 2011-08-01

**Authors:** Zakieh Rostamzadeh Khameneh, Nariman Sepehrvand, Sima Masudi

**Affiliations:** 1 Department of Microbiology and Immunology, Urmia University of Medical Sciences, Urmia, IR Iran; 2 Students’ Research Committee, Deputy for Research Affairs, Urmia University of Medical Sciences, Urmia, IR Iran; 3 Deputy for Research Affairs, Shahid Beheshti University of Medical Sciences, Tehran, IR Iran

**Keywords:** Hepatitis E, ELISA, Anti-HEV IgG, Renal transplantation

## Abstract

**Background:**

Renal transplant recipients are known to be susceptible to viral infections, with more severe clinical presentations compared to healthy persons. Hepatitis E is generally a self-limited disease, which is caused by hepatitis E virus. Recently, hepatitis E has become more important in organ transplant recipients, because of new findings regarding the potential for chronic infections in this patient group.

**Objectives:**

This study aimed to evaluate the seroprevalence of anti-HEV IgG among kidney transplant recipients in Urmia, in the north-western region of Iran.

**Patients and Methods:**

91 patients were selected randomly from amongst patients who had undergone kidney transplantation in Urmia, Iran. Each patient was tested for the presence of anti-HEV IgG antibody using an enzyme-linked immunosorbent assay (ELISA, Dia.Pro; Diagnostic Bioprobes, Italy).

**Results:**

28 subjects (30.8%) were seropositive for anti-HEV IgG. Seropositive patients were generally older than seronegative patients (P = 0.009). There was no correlation between HEV infection and the level of education (P = 0.206), the history of blood transfusion (P = 0.164), or history of pre-transplantation hemodialysis (P = 0.228). There was no significant difference in the serum alanine aminotransferase (ALT) levels of the anti-HEV seropositive and seronegative patients. Multinomial logistic regression analysis indicated no significant relationship between HEV infection and increase in ALT levels, even after controlling for treatment with azathioprine (P = 0.79, OR = 1.12; 95% CI: 0.45–2.76).

**Conclusion:**

The anti-HEV IgG antibody has a high prevalence in Iranian kidney transplant recipients, and it is significantly higher in comparison with previous studies in the general population or in hemodialysis patients.

## 1. Background

Hepatitis E is generally a self-limited disease, which is caused by hepatitis E virus (HEV) [1][2], a non-enveloped single-stranded RNA virus [3]. It is an endemic disease in developing countries in Central Asia, the Middle East, Africa, and Latin America [4], and appears to be an emerging disease in industrialized countries [5]. Iran, in the Middle East, is one of the endemic countries for HEV [6], with a few suspected outbreaks of HEV infection [7]. The seroprevalence of anti-HEV IgG in healthy Iranian populations was demonstrated to be different across regions of Iran, ranging from 2.3% in the northern region to 11.5% in southern Iran (with a cumulative rate of 153/2900 subjects; 5.2%) [8][9][10][11][12]. Hepatitis E virus has been reported to have four different genotypes, of which genotype 1 is the most prevalent type and is widespread in endemic regions, including most parts of Asia, including Iran, and northern Africa [13]. The genotype of HEV causing HEV infections in developed countries is mostly different from that in endemic regions.

The main route of HEV transmission is the fecal-oral route. However, several studies have also emphasized that transmission of HEV via non-fecal-oral routes appears to be possible [2]. There are reports of HEV infection in individuals who received blood transfusions in endemic areas [14][15][16]. HEV infection was thought to be an acute infection, which does not become chronic, with spontaneous recovery in almost all cases [1]. However, several cases of persistent (chronic) HEV infection and its relatively rapid evolution to HEV-related cirrhosis have been reported in organ transplant recipients [17][18][19]. Previous seroprevalence studies found that 6% to 15.6% of renal transplant recipients were positive for anti-HEV IgG antibodies [5][20][21].

## 2. Objectives

Considering the differences in natural history of HEV infection in organ transplant recipients compared to other patient groups, and also different prevalence rates among developing and industrialized countries, this study aimed to evaluate the seroprevalence of anti-HEV IgG among kidney transplant recipients in Urmia, Iran.

## 3. Patients and Methods

The current descriptive, cross-sectional study was conducted with the approval of the Scientific and Ethical Review Board of Urmia University of Medical Sciences (UMSU), in Urmia, Iran. 91 patients were selected randomly from amongst patients who had undergone kidney transplantation between 1991 and 2010 in the Department of Transplantation, Imam-Khomeini Training Hospital (almost 2000 cases), and referred to the Transplantation Clinic for follow-up. Informed consent was obtained from each patient prior to participation in the study. Two milliliter blood samples were obtained via venipuncture for serological analyses. Samples were centrifuged and sera were separated immediately. Sera were stored at -20ºC, and tested for the presence of anti-HEV IgG antibody by enzyme-linked immunosorbent assay (ELISA, Dia.Pro; Diagnostic Bioprobes, Italy). The presence of anti-HEV IgG antibody was considered as the evidence for prior exposure to HEV [6].

All patients had been tested previously for HBsAg (Diasorin, USA), anti-HCV (Diasorin, USA), and anti-HIV (Biotest, Germany) using ELISAs. Blood samples were evaluated for alanine aminotransferase (ALT) levels using a Hitachi autoanalyzer 704 (Roche, Switzerland) with Pars Azmoon reagents kit (Tehran, Iran). Alanine aminotransferase levels greater than 1.5 times the upper normal limit were considered as elevated ALT levels. Data were collected regarding the following variables: age, sex, educational status, marital status, etiology of end-stage renal disease (ESRD), duration of ESRD, history of hemodialysis (HD), history of blood transfusion and immunosuppressive therapy.

All collected data were analyzed using SPSS software Ver. 16 (Chicago, IL). Descriptive statistics were reported as the mean ± SD for continuous variables and as the frequency (%) for dichotomous variables. To evaluate the relationship between different factors, we performed chi-square analysis. Quantitative variables were compared using independent t-test. P < 0.05 were considered statistically significant.

## 4. Results

Ninety-one renal transplant recipients were selected randomly from recipients who had undergone kidney transplantation between 1991 and 2010 in the Imam Khomeini Hospital in Urmia and they were enrolled in the study after giving informed consent for blood sampling and laboratory assays. The mean age of the patients was 35.4 ± 14.5 years old (6-65 years old). 61 patients (67%) were men and the remaining (33%) were women. 59 (64.8%) were married and 32 patients (35.2%) were single. 19 patients (20.9%) were illiterate, 20 (22%) had studied until the elementary level, 10 (11%) attained guidance school level, 10 (11%) attained high school level, 31 (34%) had diplomas, and one (1.1%) patient had an academic degree.

The cause of renal failure among the patients was glomerulonephritis in 30 cases (33%),; hypertension in 28 cases (30.8%),; polycystic kidney disease (PCKD) in 12 cases (13.2%); renal atrophy in 3 cases; nephrolithiasis and focal-segmental glomerulosclerosis (FSGS) in 2 cases; and DM, Alport syndrome, neurogenic bladder, and urinary infection each in one (1.1%) of the participants. 65 patients (71.4%) had undergone HD prior to renal transplantation, but 26 (28.6%) had no history of HD prior to transplantation. Only 37 subjects (40.7%) had a history of blood transfusion. Mean anti-HEV IgG titer was 1.47 ± 2.16 (range, 0.14-7.31). 28 subjects (30.8%) were seropositive for anti-HEV IgG; however, 59 cases (64.8%) were seronegative, and 4 subjects (4.4%) had a borderline IgG titer (range, 0.9-1.1). We categorized patients with borderline anti-HEV titer as seronegative; accordingly, seronegative cases were increased to 63 cases (69.2%). None of the participants, either in the anti-HEV positive group or in the seronegative group, were seropositive for blood-borne viruses (hepatitis B virus [HBV], hepatitis C virus [HCV], or human immunodeficiency virus [HIV]). There was no statistically significant association between HEV seropositivity and the other viruses mentioned above.

A comparison of patient characteristics between the groups seropositive and seronegative for anti-HEV IgG is shown in Table 1. Seropositive patients were generally older than seronegative patients (P = 0.009). In this study, we did not find a significant correlation between HEV infection and level of education (P = 0.206) (Table 1). There was no correlation between a history of blood transfusion and HEV infection (P = 0.164). Furthermore, chi-square analysis revealed no correlation between a history of pre-transplantation hemodialysis and HEV infection (P = 0.228). The mean ALT level was 68.5 ± 35.4 units per liter (IU/L), when all patients were included in the calculation of the mean (27-262). The mean aspartate aminotransferase (AST) level was 51.4 ± 18.4 IU/L. The mean ALT level was 1.71 times the normal values. Fifty patients (54.9%) had normal ALT levels, but 41 patients (45.1%) had elevated ALT levels.

**Table 1:** Patient Characteristics of Two Different Groups, Positive, or Negative, for the Anti-HEV IGg Antibody

Characteristic	**Anti-HEV Positive (n = 28)**	**Anti-HEV Negative (n = 63)**	***P* value**
Age, y, mean ± SD	41.3 ± 12.4	32.8 ± 14.6	0.009
Sex, No. (%)			0.366
Male	20 (71.4%)	41 (65.1%)	
Female	8 (28.6%)	22 (34.9%)	
Education, No. (%)			0.206
Illiterate	9 (32.1%)	10 (15.9%)	
Lower than diploma	11 (39.3%)	29 (46%)	
Diploma and higher	8 (28.6%)	24 (38.1%)	
History of blood transfusion, No. (%)			0.164
Positive	14 (50%)	23 (36.5%)	
Negative	14 (50%)	40 (63.5%)	
History of HD, No. (%)			0.228
Positive	22 (78.6%)	43 (68.3%)	
Negative	6 (21.4%)	20 (31.4%)	
ALT levels, No. (%)			0.48
Normal	16 (57.1%)	34 (54%)	
Elevated	12 (42.9%)	29 (46%)	
ALT, IU/L, mean ± SD	65.1 ± 40.7	70.0 ± 33.1	0.550
AST, IU/L, mean ± SD	47.9 ± 10.1	53.0 ± 21.0	0.229

Chi-square analysis revealed no relationship between treatment with azathioprine and increase in liver enzymes (ALT) (Table 2). Further, there was no significant difference in the serum ALT level between anti-HEV seropositive and seronegative patients. Multinomial logistic regression indicated no significant relationship between HEV infections and increases in ALT levels, even after controlling for treatment with azathioprine (P = 0.79, OR = 1.12; 95% CI: 0.45-2.76).

**Table 2:** Alanine Aminotransferase Levels among Patient Groups with or without Azathioprine in Their Immunosuppressive Regimen

Characteristic	**Treatment with Azathioprine, No. **(%) **(n = 30)**	**Not Treated with Azathioprine, No. **(%)** (n = 61)**	***P* value**
Normal ALT level	18 (60%)	32 (52.5%)	0.325
Elevated ALT level	12 (40%)	29 (47.5%)	

## 5. Discussion

Renal transplant recipients are known to be susceptible to viral infections, with more severe clinical presentations compared to healthy persons [13][22]. The increased susceptibility is due to immunosuppression, which is caused by treatment with immunosuppressive drugs. Recently, hepatitis E has become more important in organ transplant recipients. To date, hepatitis E had been believed to be a self-limited acute infection, which rarely becomes chronic. However, in recent years, some articles have reported HEV-related chronic hepatitis, and even cirrhosis, in organ transplant recipients [5][17][18][23][24]. Since persistent HEV viremia was also observed in patients with T-cell lymphoma while receiving chemotherapy [25], the cause of this chronicity could be immunosuppression, which is similar in both patient groups.

Other studies have questioned the generalizability of these findings (chronicity of HEV infection in organ transplant recipients), since all the studies that described chronic infection or cirrhosis in renal transplant patients reported infections with genotype 3 HEV, when viral genotyping was performed. However, genotype 3 is responsible for only a minor proportion of acute hepatitis E cases around the world [6][13], and has different clinical features compared to genotype 1 HEV. It seems to be a zoonotic infection in humans, whilst genotype 1, which is the most prevalent genotype of HEV in the world, is mostly transmitted via the fecal-oral route. Iran, located in the Middle East, is a country with few suspected outbreaks of HEV [7]. A population-based study in Iran reported the prevalence rate of anti-HEV IgG among the healthy population to be 9.6% [12]. The prevalence rate among hemodialysis patients was reported to be 7.4% in Tabriz, Iran [4]. Therefore, the prevalence rate was lower than that in the general population. The differences were attributed to differences between the populations, the sample size, or the situation of public health services [4].

Hoseini-Moghaddam et al. stated that the seroprevalence of HEV in patient groups, such as HD patients, seems to be dependent on the prevalence of HEV infection in the general population [2]. Kamar et al. reported the seroprevalence of HEV in French renal transplant recipients to be 14.5% [5], whilst its prevalence in blood donors in southwestern France, represented as the general population, was 16.6% [26]. Our study revealed the seroprevalence of anti-HEV IgG to be significantly higher in transplant recipients than in the Iranian general population, or in HD patients. Therefore, we conclude that the prevalence rate of anti-HEV IgG in transplant recipients is independent of its prevalence in the general population. The seroprevalence of anti-HEV IgG among Iranian transplant recipients reported here is the highest reported in the world in similar populations (2.7%-15.6%) [1][5][20][21]. The high seroprevalence is even more interesting, considering the lower prevalence rate of HEV in the Iranian general population than in most countries in which HEV is endemic.

It is possible that this high seroprevalence of HEV is due to infection with another unknown virus, which creates antibodies cross-reacting with HEV [27]. The study of Kamar et al. determined the clinical importance of acute hepatitis E infection in organ transplant recipients, despite all previous beliefs that considered Hepatitis E infection as a self-limited disease. Among 14 transplant recipients with acute hepatitis E infection, confirmed by elevated liver enzymes and positive HEV RNA tests, 8 patients (57%) developed chronic hepatitis. The patient's age was the only factor that was indicated to have a correlation with HEV infection. Seropositive patients were generally older than seronegative patients (P = 0.009).

Moreover, the relatively rapid evolution to cirrhosis has been mentioned by some authors, particularly in the setting of kidney transplantation [18][19]. Further, Kamar et al. suggested that HEV infection in kidney transplant recipients is more severe that HCV infection [17][28]. Some studies have proposed blood transfusion as a potential route of HEV transmission, particularly in areas of high endemicity [14][15][29]. However, in this study, we did not find any correlation between a history of blood transfusion and HEV infection. Certain drugs are also considered a probable cause of an increase in liver enzymes, when other frequent causes have been ruled out. Hepatotoxic features have been reported in 2 immunosuppressive drugs, azathioprine [30] and sirolimus [31].

In our study, chi-square analysis revealed no relationship between treatment with azathioprine and increase in liver enzymes (ALT). Further, there was no significant difference in the serum ALT levels of anti-HEV seropositive and seronegative patients. Multinomial logistic regression indicated no significant relationship between HEV infection and increase in ALT levels, even after controlling for treatment with azathioprine. In this study, 12 (42.9%) cases of 28 seropositive recipients had high ALT levels. This is in accordance with previous findings by Kamar et al. in France (14 of 46 seropositive cases: 35%) [5]. The overall prevalence of individuals with abnormal ALT in general population is demonstrated to be 11%-13%. In 25%-35% of individuals with abnormal ALT, the probable cause is nonalcoholic fatty liver disease [32][33]. In this study, the prevalence rate of abnormal ALT level in the HEV seronegative patients was 46%, which is considerably higher than the rates reported in the general population. However, in our study the cases and controls were both transplant recipients, who have their own risk factors (i.e., the immunosuppressive treatment) for liver function impairment.

We did not find a significant correlation between HEV infection and the level of education. This was in accordance with the study of Mathur et al. [34], where the prevalence of HEV was found to be lower in rural areas, with high number of uneducated people than in urban areas, but in contrast to the study of HEV risk factors among pregnant women in Turkey [35]. However, since the design of our study was not suitable for comparing the anti-HEV antibody among different patient groups (different sexes, educational status, history of blood transfusion etc.), we cannot rely on P-values completely, and the results should be interpreted cautiously. In our study, none of the participants were seropositive for blood-borne viruses (HBV, HCV, and HIV). Consequently, there was no statistically significant association between HEV seropositivity and the other viruses mentioned above. This was in accordance with the findings of another study from Iran among HD patients [4], but in contrast to the findings of Pisanti et al. from Italy, which reported an association between hepatitis C and HEV infection [36].

We did not evaluate all the samples or even the seropositive cases for serum anti-HEV IgM or HEV RNA. Therefore, we cannot draw any conclusions regarding the timing of exposure, prior or recent, of our seropositive patients to HEV. Since we did not investigate the subjects for anti-HEV IgM or HEV RNA, any conclusions regarding the correlation between anti-HEV IgG and elevated ALT levels cannot be further confirmed. Further, from our data, we cannot discuss the acuteness or chronicity of HEV infection; first, because of the lack of information regarding anti-HEV IgM or HEV RNA, and second, due to the cross-sectional design of the study. As mentioned before, the most common route of transmission for HEV is the fecal-oral route [2][6]. In this study we did not identify any source for HEV contamination. Nonetheless, considering the fecal-oral route as the main route of transmission in endemic regions, we anticipate that the virus was transmitted via water and food intake.

Several HEV vaccines have been produced to date, but they have not reached the market yet [6]. Furthermore, cost-effectiveness analysis is needed to provide proper advice regarding the appropriateness of anti-HEV vaccination in transplant recipients prior to transplantation. Several studies have demonstrated that chronic viral hepatitis E could be a silent disease in graft recipients [5]. The authors of these studies suggested that clinicians should not look merely for clinical evidence to consider hepatitis E. Chronic patients may have normal liver enzymes or remain seronegative [2]; therefore, long-term follow-up of the patients is recommended. The anti-HEV IgG antibody has a high prevalence in Iranian kidney transplant recipients, and it is significantly higher in comparison with that in previous studies in the general population or HD patients.
